# The roles of extracellular vesicles in mental disorders: information carriers, biomarkers, therapeutic agents

**DOI:** 10.3389/fphar.2025.1591469

**Published:** 2025-04-09

**Authors:** Longkun Wang, Ruixue Liu, Ying Wang

**Affiliations:** Department of Pharmacy, Tianjin Anding Hospital, Tianjin, China

**Keywords:** extracellular vesicles, mental disorders, biomarkers, information crosstalk, therapy

## Abstract

Mental disorders are complex conditions that encompass various symptoms and types, affecting approximately 1 in 8 people globally. They place a significant burden on both families and society as a whole. So far, the etiology of mental disorders remains poorly understood, making diagnosis and treatment particularly challenging. Extracellular vesicles (EVs) are nanoscale particles produced by cells and released into the extracellular space. They contain bioactive molecules including nucleotides, proteins, lipids, and metabolites, which can mediate intercellular communication and are involved in various physiological and pathological processes. Recent studies have shown that EVs are closely linked to mental disorders like schizophrenia, major depressive disorder, and bipolar disorder, playing a key role in their development, diagnosis, prognosis, and treatment. Therefore, based on recent research findings, this paper aims to describe the roles of EVs in mental disorders and summarize their potential applications in diagnosis and treatment, providing new ideas for the future clinical transformation and application of EVs.

## Graphical Abstract

**Figure:**
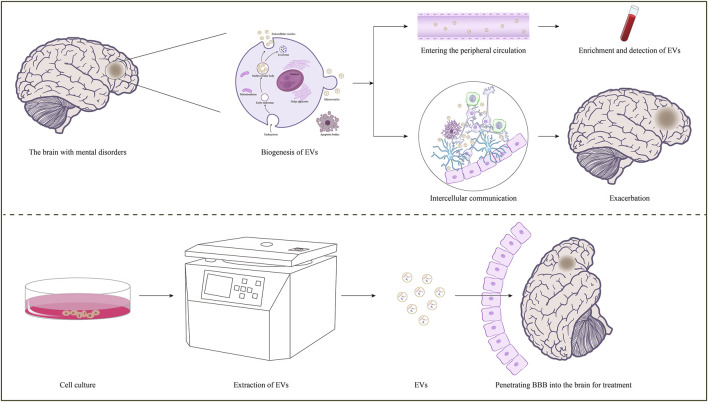
The roles of EVs on mental disorder. In the brain with mental disorders, some brain cells-derived EVs can cross the BBB into the peripheral circulation, and specific information related to the disease can be obtained through enrichment and detection. Moreover, some brain cells-derived EVs are involved in intercellular communication, transmitting pathological information and regulating disease progression.

## 1 Introduction

Mental disorders are a range of diseases characterized by cognitive, thinking, emotional, or behavioral abnormalities, that may arise from unfortunate experiences or impairment of vital functional areas (Wu et al., 2023; Louka and Koumandou, 2024). According to its pathological mechanism and clinical manifestations, mental disorders can be divided into many different types, the most common are schizophrenia (SCZ), major depression disorder (MDD), bipolar disorder (BD), and so on (Collaborators, 2022; Guo et al., 2024). In recent years, the burden caused by mental disorders has been increasing globally, presenting significant challenges to public health and social security (Kieling et al., 2024; The Lancet, 2024). Based on data from the Global Burden of Disease Study 2019, about 1 billion people worldwide suffer from mental illness, resulting in 125.3 million disability-adjusted life years (DALYs) (Deng et al., 2024; Arias et al., 2022; Guo et al., 2024). Mental disorders are the leading cause of disability worldly, contributing to personal dysfunction and reduced quality of life. In addition, patients with mental disorders may be accompanied by various complications, causing the deterioration of their condition and premature death (Merrill and Ashton, 2024). However, the etiology and pathogenesis of mental disorders are complex and diverse, the symptoms are hidden, and there are difficulties and challenges in diagnosis and treatment (Pullman et al., 2021). Therefore, it is essential to explore specific biomarkers and safe and effective therapeutic drugs to provide new strategies for clinical application.

**FIGURE 1:**
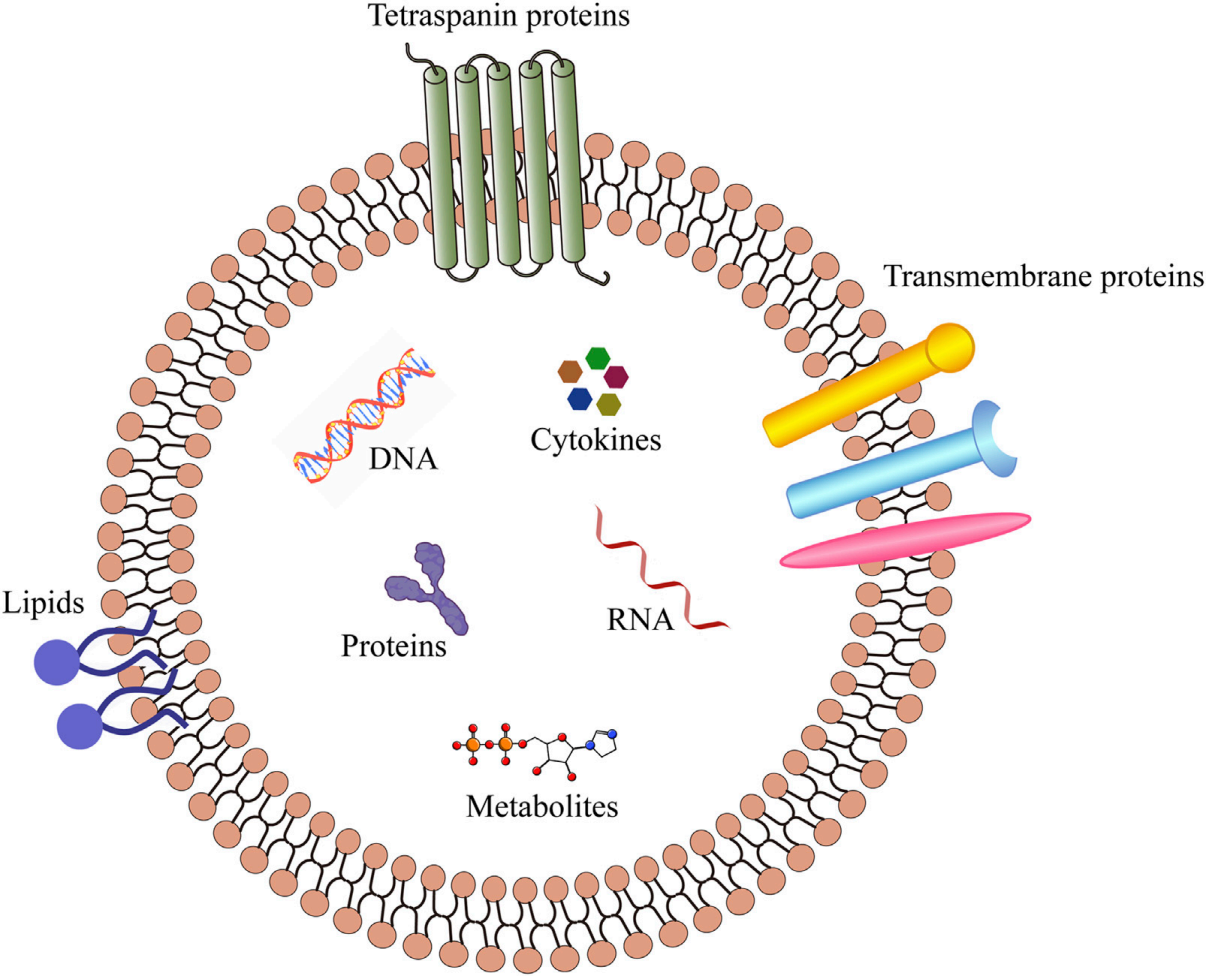
Structure of EVs.

**FIGURE 2:**
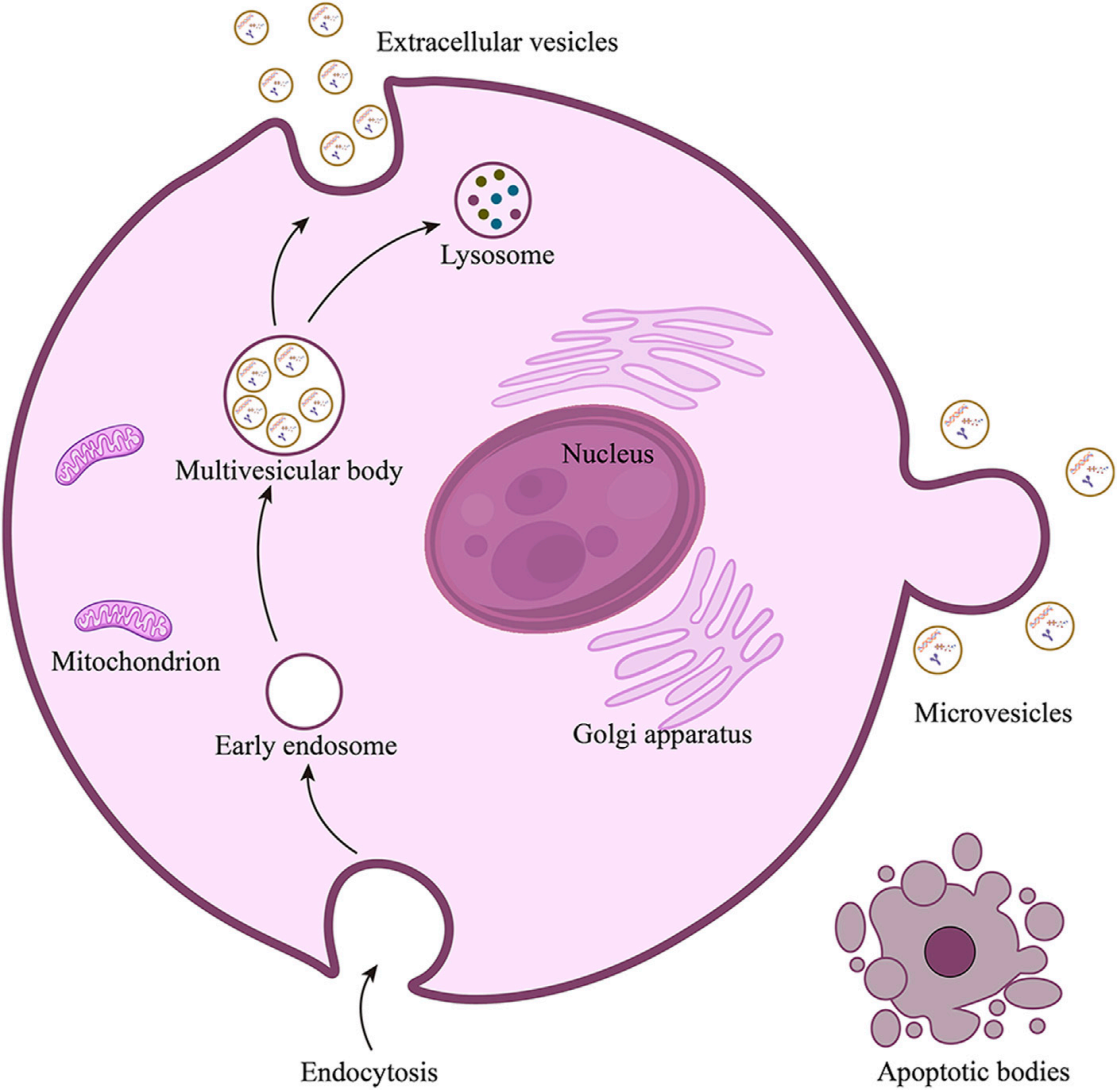
Biogenesis of EVs.

Minimal information for studies of extracellular vesicles (MISEV 2023) defines the extracellular vesicles (EVs, Figure 1) as particles secreted by cells and released into the extracellular space, which are composed of lipid bilayers and cannot be replicated by themselves (Welsh et al., 2024). According to the differences in source, biogenesis, preparation methods, composition, and size, EVs can be divided into several subtypes, including exosomes, microvesicles, and apoptotic bodies (Figure 2). Exosomes are vesicles derived from endocytosis, with a particle size of 30–150 nm, which are released by the endolysosomal pathway after the fusion of multivesicular bodies with the plasma membranes. Microvesicles are outward vesicles originating from plasma membrane budding, with a particle size of 100–1,000 nm, larger than exosomes. Apoptotic bodies are vesicles that originate from apoptotic cells and are released during apoptosis, with a particle size between 200 and 1,000 nm. However, there is still no standardized nomenclature for different types of EVs, the prefix “operable term” of EVs needs to be used with caution (Théry et al., 2018; Welsh et al., 2024). EVs are ubiquitous in all biological fluids and contain various biological components such as proteins, lipids, DNA, RNA and cytokines. They play an important role in intercellular information transmission and are widely involved in numerous physiological and pathological processes (van Niel et al., 2022; Sung et al., 2021; Liu and Wang, 2023). Changes in the contents of EVs reflect changes in the physiological microenvironment, suggesting that EVs can be used as promising biomarkers to provide new insights into the diagnosis and treatment of several diseases.

In the central nervous system (CNS), EVs can be secreted and taken up by neurons, microglia, astrocytes, oligodendrocytes, endothelial cells and other cells to mediate information exchange between cells, and then regulate nerve development, regeneration, synaptic function and so on (Filannino et al., 2024; Bahram, 2021; Zhang et al., 2022). Furthermore, a growing number of studies have demonstrated that small-sized EVs can cross the blood-brain barrier (BBB) bidirectionally, playing a crucial role in communication between the peripheral circulation and the CNS (Ramos-Zaldívar et al., 2022; Liang et al., 2024; Xu X. et al., 2024; Bhom et al., 2025). Consequently, EVs derived from brain cells may carry abnormal information into the peripheral circulation and can be used for diagnosing mental disorders. Moreover, given the advantages of EVs, including low immunogenicity, high stability, biocompatibility, modifiability, and the ability to cross biological barriers, multiple EVs-related treatment systems have been established (Yang et al., 2024). In recent years, researchers have devoted plenty of effort to the research field of EVs-related therapies for CNS diseases, and the results have also given positive feedback. For instance, mesenchymal stem cells-derived EVs can promote recovery from traumatic brain injury by protecting and repairing nerves (Xiong et al., 2024); engineered mesenchymal stem cells-derived EVs can improve cognitive function by eliminating abnormal protein accumulation and regulating the immune response (Yin et al., 2023). Hence, EVs can deliver abundant bioactive molecules into the brain and regulate various biological processes to exert therapeutic potential in mental disorders.

**FIGURE 3:**
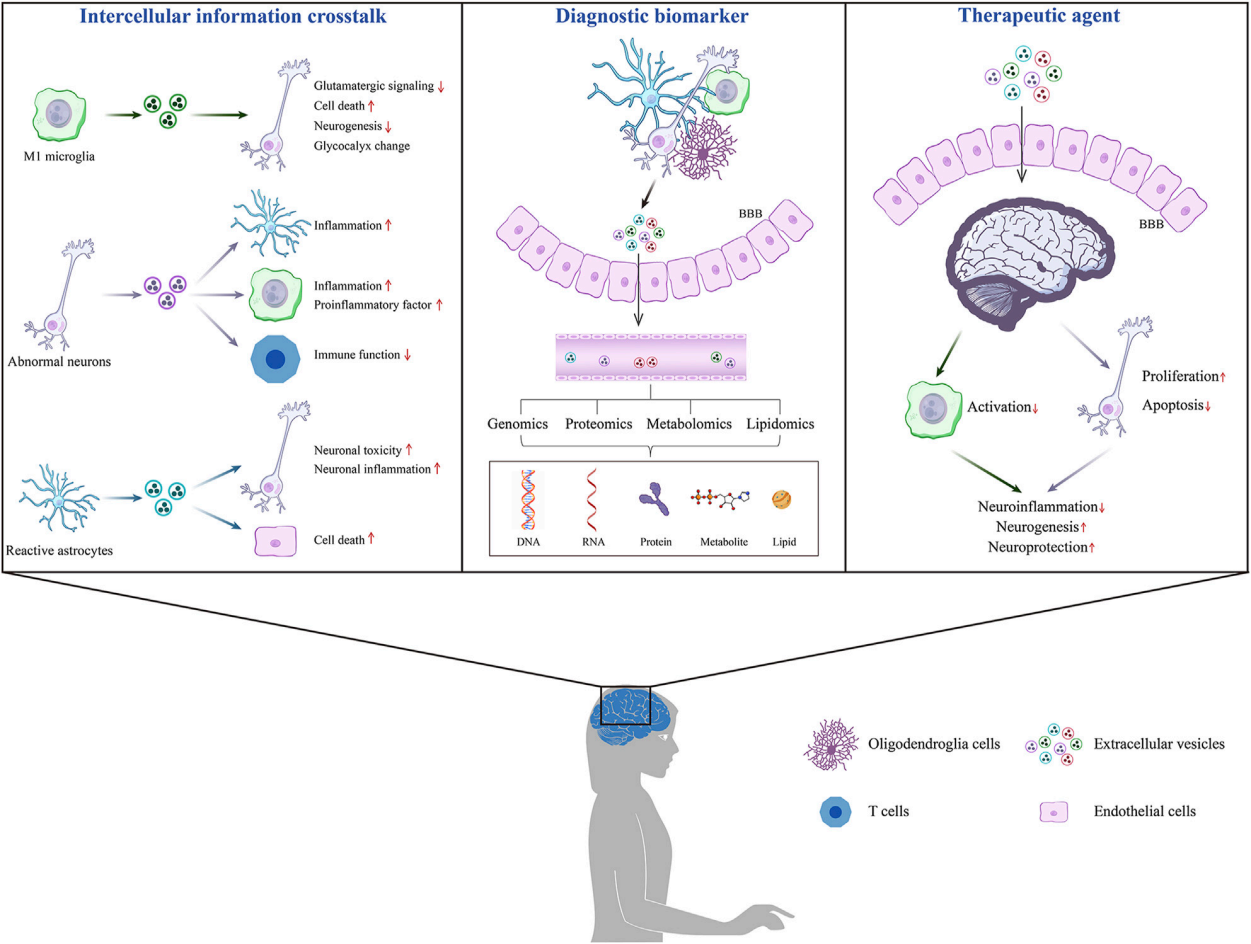
The effects of EVs on mental disorders.

This paper describes the roles of EVs in common mental disorders and explores their potential as biomarkers and therapeutic molecules, providing a theoretical foundation for future clinical applications. This article is divided into three sections: EVs as information carriers for intercellular communication, EVs as potential biomarkers for diagnosing mental disorders, and the application of EVs in the treatment of mental disorders (Figure 3).

## 2 EVs as information carriers for intercellular communication

In the CNS, communication crosstalk is prevalent between different cells and is essential for neural development and homeostasis (Liu et al., 2023; Farizatto and Baldwin, 2023; Szepesi et al., 2018). Bidirectional communication between cells is usually mediated by neurotransmitters, ions, EVs, etc., which is vital for various biological functions such as synaptic transmission, inflammation regulation, axon integrity, and neural circuit maturation (Wilton et al., 2019; Cserép et al., 2021). As important information carriers for complex signal transmission between brain cells, EVs have recently received extensive attention in research related to mental disorders (Lizarraga-Valderrama and Sheridan, 2021; Filannino et al., 2024; Ahmad et al., 2022).

### 2.1 EVs derived from microglia

Microglia, a type of glial cells, function as macrophages in the CNS, accounting for about 5%–12% of the total cells in the brain and spinal cord (Woodburn et al., 2021). They are widely distributed in the brain and are mainly involved in processes such as cell debris removal, synaptic pruning, inflammation regulation, and homeostasis monitoring (Cornell et al., 2022). Microglia can exist in different states depending on the type and duration of stimulation they receive. When transiently stimulated, microglia can protect the brain. However, continuous stimulation can cause damage to the normal brain. These processes are mediated by the interaction between microglia with other brain cells, such as neurons and astrocytes through direct or indirect contact (Rahimian et al., 2021; Li et al., 2022; Wang H. et al., 2022). Chronic neuroinflammation is a common feature of mental disorders, resulting in sustained activation of microglia. This ongoing activation can cause the secretion of abnormal EVs by microglia, which have effects in regulating disease progression (Rahimian et al., 2021).

It is speculated that the low glutamatergic signal transduction function of N-methyl-D-aspartate receptors (NMDARs) and the excessive activation of dopamine D2 receptors may promote the progression of schizophrenia (Borroto-Escuela et al., 2016; Balu, 2016). Borroto-Escuela et al. found that under mild inflammatory conditions, EVs containing recombinant chemokine C-C-motif receptor 2 (CCR2), C-X-C chemokine receptor 4 (CXCR4), and recombinant interleukin 1 receptor type II (IL1R2), along with their ligands, are secreted by microglia and engulfed by neurons. CCR2, CXCR4 and IL1R2 can form heteroreceptor complexes with NMDAR in the cell membrane, leading to pathological allosteric receptor complexes and reducing normal glutamatergic signal transduction. Moreover, they can create heteroreceptor complexes with dopamine D2 receptors, increasing D2 receptor activation. As a result, EVs secreted by microglia transmit signals to neurons through NMDARs and D2 pathways, exacerbating schizophrenia-like symptoms (Borroto-Escuela et al., 2017). In the case of degenerative diseases, persistent DNA damage leads to the accumulation of double-stranded DNA (dsDNA) fragments in microglia and the release of dsDNA-containing EVs. Arvanitaki et al. detected that these EVs are transported to neurons, triggering the death of neurons and accelerating the occurrence of neurodegenerative symptoms (Arvanitaki et al., 2024). Studies have shown neuronal α-synuclein is a pathological protein, leading to cognitive impairment. Guo et al. studied that EVs extracted from cerebrospinal fluid microglia of patients with Parkinson’s disease (PD) contain α-synuclein oligomers, which can infect neurons and induce α-synuclein aggregation in neurons, causing mental disorders in PD patients (Guo M. et al., 2020). Furthermore, Fan et al. revealed that in depressed mice, miR-146a-5p was enriched in EVs secreted by microglia, and then shuttled to neurons to inhibit neurogenesis by directly targeting kruppel-like factor 4 (KLF4) (Fan et al., 2022). In addition, microglia can transfer neurotropin-3 (Neu3) to neurons through EVs to regulate the remodeling of neuronal glycocalyx, thus affecting the connections between neurons (Delaveris et al., 2023).

To sum up, microglia-derived EVs are involved in the pathological progression of mental disorders by regulating different signaling pathways. However, in the future, more extensive and comprehensive studies on microglia-derived EVs are needed to provide a more solid theoretical basis for systematically elucidating their roles in the progression of mental disorders.

### 2.2 EVs derived from neurons

Neurons are the basic structural and functional units of the CNS, which are responsible for receiving, integrating, and transmitting information to regulate complex neural activities (Jeon and Kim, 2023). In a healthy brain, neurons can transmit information through EVs to influence the proliferation, differentiation, and polarization of brain cells, playing an important role in various physiological processes. Studies have demonstrated that EVs secreted by neurons can transport vascular endothelial growth factor A (VEGF-A) to endothelial cells, promoting their proliferation and mediating angiogenesis and maturation (Dong et al., 2022). In addition, neurogenic EVs were isolated from the culture supernatant of rat cortical neurons and subsequently introduced to the co-culture of primary rat microglia. The results showed that neuron-derived EVs could reduce the expression of pro-inflammatory cytokines such as tumor necrosis factor-α (TNF-α), interleukin-6 (IL-6), monocyte chemoattractant protein-1 (MCP-1), and the gene inducible nitric oxide synthase (iNOS). This activity mitigated the lipopolysaccharide (LPS)-induced pro-inflammatory response in microglia, thereby achieving the purpose of controlling the inflammatory balance (Peng et al., 2021).

However, in the diseased brain, dysfunctional neurons can also transmit pathological information through EVs, altering the normal function of other cells, inducing increased neuroinflammation, and causing a vicious circle (Jiao et al., 2022). As mentioned above, Kaya et al. have found that after stimulation, EVs containing high mobility group box-1 protein (HMGB-1) can be released by stressed neurons. These EVs were then transported to astrocytes, activating the toll-like receptor 4 (TLR4) signaling pathway, thereby initiating the inflammatory signal of astrocytes (Kaya et al., 2023). In addition, in a mouse model of depression, Xian et al. found that neuron-derived EVs are rich in miRNA-9-5p, which can activate microglia by inhibiting suppressors of cytokine signaling 2 (SOCS2) expression and activating the JAK/STAT3 pathway to promote the release of inflammatory factors, thus exacerbating cognitive and behavioral disorders in mice (Xian et al., 2022). Durur et al. isolated neuron-derived EVs (sNDEVs) from healthy volunteers and Alzheimer’s disease (AD) patients. The dysregulated expression of miRNA let-7e in sNDEVs of AD patients was identified by sequencing technology and qRT-PCR. After co-culture of miRNA Let-7E-rich sNDEVs with microglia, it was found that the expression of the interleukin-6 (IL-6) gene in microglia was significantly increased, suggesting that sNDEVs from neurons of AD patients may promote the inflammatory response of microglia (Durur et al., 2022). Moreover, it has been found that neuronal EVs in disease states can cause extensive inhibition of T cells. These EVs can not only inhibit the differentiation of naive CD4^+^ T cells into Th1 and Th2 cells but also induce the expression of PD-L1 on T cells, inhibiting the function of T cells together (Chen et al., 2024).

In the state of mental illness, neurons can become damaged, and the abnormal secretion of EVs is involved in regulating neuroinflammation, which aggravates the progression of the disease. Therefore, the study of neuron-derived EVs may provide new insights into the diagnosis and treatment of psychiatric disorders. However, the current relevant research in this area is not systematic, and more attention needs to be paid in the future to advance our understanding of this field.

### 2.3 EVs derived from astrocytes

Astrocytes are important regulatory and supportive cells in the CNS that play a vital role in system homeostasis (Liu et al., 2021). It has been demonstrated that astrocyte-derived EVs support the differentiation of oligodendrocytes and promote myelin generation, which is crucial for maintaining neuronal axon function (Willis et al., 2020). In addition, astrocyte-derived EVs can also regulate the communication between glial cells and neurons by affecting the phenotypic changes of neuronal cells (You et al., 2020). Therefore, EVs derived from glial cells can participate in the development, metabolism and energy supply of neurons through various ways to maintain the normal function of neurons (Zhang et al., 2021). However, in the context of neurological diseases, astrocyte-derived EVs can negatively impact the normal function of neurons through multiple pathways, leading to increased disease symptoms (Xu H. et al., 2024). One classic pathological feature of AD is the accumulation of TAU protein. Richetin et al. have discovered that TAU protein accumulates in astrocytes of AD model mice and is released into the cell in the form of EVs, then transported to neurons to accumulate, inducing neuronal toxicity and causing cognitive and functional impairment in patients (Richetin et al., 2020). Moreover, sphingolipid ceramide (Cer) is upregulated in the pathological brain, which can stimulate microglia to release pro-inflammatory factors such as C1q, TNF-α and interleukin-1α (IL-1α) (Filippov et al., 2012). Subsequently, in response to these pro-inflammatory factors, reactive astrocytes can produce Cer-riched EVs, which are absorbed by neurons and induce mitochondrial-dependent apoptosis, further aggravating neuroinflammation (Crivelli et al., 2023). In addition to neurons, astrocyte-derived EVs can also affect other cells. For example, Gonzalez-Molina et al. found that EVs secreted by astrocytes targeted neurovessels, inducing endothelial cell damage and cerebrovascular deterioration (González-Molina et al., 2021). Therefore, given these complex interactions in the system, more in-depth research and exploration of astrocyte-derived EVs is warranted, which may be valuable for the remission of mental disorders.

In summary, after the occurrence of mental disorders, the brain microenvironment changes, and various abnormal brain cells secrete EVs containing pathological information into the circulation. As carriers of intercellular communication, these EVs interact with parental and non-parental cells in different ways and participate in the regulation of disease progression. In addition, these EVs reflect the physiological state of the parent cells to a large extent, which provides a possibility for predicting the physiological and pathological state of the brain.

## 3 EVs as potential biomarkers for diagnosing mental disorders

In recent years, there has been growing interest in the potential of EVs as biomarkers for disease diagnosis (Schou et al., 2020; Liang et al., 2021; Kong et al., 2023). Almost all cell types can release EVs, which contain functional biomolecules that reflect the physiological information of parental cells. In addition, CNS-derived EVs can cross the BBB and exist stably in the peripheral circulation (Xu X. et al., 2024). Compared with collecting cerebrospinal fluid, extraction of EVs from peripheral blood is less invasive and more convenient (Zhang X. M. et al., 2024). At present, biomarkers for the diagnosis and monitoring of mental disorders mainly include immune factors and neurotransmitters. However, both may change in various brain diseases, rather than specifically targeting a specific disease. EVs biomarkers identified according to the disease type may be highly sensitive and specific, contributing to early disease diagnosis (Kong et al., 2023). In addition, EVs possess stable lipid bilayer membranes that can protect the contained active substances from degradation, providing more comprehensive information for disease diagnosis. Therefore, EVs are considered attractive candidates as biomarkers that can reflect the state of the brain microenvironment, providing valuable insights into the diagnosis of mental disorders.

### 3.1 Schizophrenia (SCZ)

SCZ is a chronic and common mental disorder with high morbidity and disability, which brings a huge burden to families and society (McCutcheon et al., 2020; Jauhar et al., 2022). It is a heterogeneous disease of diverse etiology, characterized by positive symptoms (delusions, hallucinations), negative symptoms (apathy, social withdrawal, anhedonia), and cognitive impairment (Faden and Citrome, 2023). Currently, clinical diagnosis of SCZ is primarily based on operational diagnostic criteria, including the Diagnostic and Statistical Manual of Mental Disorders (DSM) and the International Classification of Diseases (ICD) (Gaebel et al., 2020). However, the diagnostic form relies on a relatively subjective assessment of the patient’s symptoms, which can easily lead to diagnostic errors and delay the treatment of the disease (Raghavan et al., 2017). Therefore, the search for objective biomarkers is of great significance for improving the diagnosis and prognosis of SCZ. In recent years, EVs have shown considerable promise as potential diagnostic tools for SCZ (Wang Y. et al., 2022; Raghavan et al., 2017).

Studies have shown that EVs-derived microRNAs (EVs-miRNAs) are involved in multiple biological and pathological processes as promising biomarkers for various diseases (Raghavan et al., 2017). Based on this, several studies have been conducted to explore the function of EVs-miRNA in SCZ. For instance, Du et al. analyzed genome-wide miRNA expression profiles in serum-derived EVs from patients experiencing first-episode drug-naive SCZ (Du et al., 2019). Compared with healthy controls, the expression of has-miR-206 was significantly upregulated in SCZ patients and induced downregulation of brain-derived neurotrophic factor, supporting the neurotrophic factor hypothesis of SCZ. Barnett et al. enriched neuron-derived EVs from the serum of SCZ patients and performed sequencing analysis. The results discovered that the expression of miRNA-1246 was significantly increased in SCZ patients, while the expression of hsa-miR-451a was continuously decreased, which is particularly important for the identification of different symptoms of SCZ (Barnett et al., 2023). Amoah et al. verified that the expression of miR-223 was significantly elevated in patients with SCZ, which was secreted in the form of EVs and then targeted glutamate receptors, playing a role in regulating neuronal function (Amoah et al., 2020). Khadimallah et al. demonstrated that the blood exosome miR-137 is elevated and cox-6a2 is decreased in patients with early psychosis (EPP). The combination of these two may guide the diagnosis of early psychosis (Khadimallah et al., 2022). Funahashi et al. determined that miR-675-3p is significantly increased in refractory SCZ and is involved in neuronal and synaptic development (Funahashi et al., 2023). Moreover, the expression of other biomolecules in EVs was also abnormal in SCZ. Du et al. conducted a metabolomics study on serum EVs in SCZ patients and identified 25 disordered metabolites, primarily related to glycerophospholipid metabolism and biosynthetic pathways of phenylalanine, tyrosine and tryptophan (Du et al., 2021). Their differential expression showed excellent performance in the diagnosis of SCZ. Xu et al. revealed notable differences in the lipid profiles of serum EVs between SCZ and healthy subjects through absolute quantitative lipidomics (Xu C. X. et al., 2024). Their findings indicated significant disturbances in sphingolipid metabolism, glycerophospholipid metabolism, and linoleic acid metabolism, which play an indispensable role in the pathophysiology of the disease. Ranganathan et al. confirmed that compared with healthy controls, the glial fibrillary acidic proteins (GFAP) from plasma EVs are significantly increased in SCZ patients, while the α-II-Spectrins are significantly decreased (Ranganathan et al., 2022). This phenomenon provides a basis for the identification of EVs as biomarkers for schizophrenia. Tunset et al. identified abnormal expression of five proteins involved in regulating glutamate synaptic plasticity in SCZ through proteomic analysis (Tunset et al., 2020). The proteins include neurogranin (NRGN), neuron-specific calcium-binding protein hippocalcin (HPCA), kalirin (KALRN), β-adducin (ADD2), and ankyrin-2 (ANK2), which supported the glutaminergic hypothesis of SCZ.

So far, some studies have explored the abnormalities of EVs in SCZ patients, demonstrating the potential value of EVs in the field of SCZ diagnosis. However, the sample size in the existing studies is limited, and further validation is needed to expand the sample size, aiming to translate the potential biomarker characteristics of EVs into the benefits of SCZ diagnosis.

### 3.2 Major depression disorder (MDD)

MDD is a type of serious mental illness and a major global health problem affecting the global population (Cui et al., 2024). Reports indicate that approximately 8.29% of individuals in China suffer from moderate to severe depression, causing a considerable health burden (Wang et al., 2024). The core symptoms of MDD are persistent low mood and loss of pleasure, which seriously affect the quality of life and social interaction of patients. Furthermore, people with MDD have a higher risk of suicide, and the World Health Organization (WHO) classifies it as the leading cause of disability globally (Gutiérrez-Rojas et al., 2020). Currently, the diagnosis of MDD largely depends on the clinical judgment of healthcare professionals and patients’ self-reports, which is easy to cause confusion in the clinical practice of MDD. Therefore, exploring more objective and rigorous biomarkers for clinical evaluation is an important challenge.

Wei et al. conducted the genome-wide analysis of miRNA expression profile in blood-derived exosomes from MDD patients and healthy volunteers. They identified one miRNA, hsa-miR-139-5p, with the most significant difference in expression, being significantly upregulated in MDD (Wei et al., 2020). The results of the receiver operating characteristic (ROC) curve showed that hsa-miR-139-5p performed excellently in distinguishing MDD patients from healthy controls, achieving an area under the curve (AUC) of 0.807, sensitivity of 0.867, and specificity of 0.767, indicating that hsa-miR-139-5p in exosomes has potential as a biomarker for diagnosing MDD (Liang J. Q. et al., 2020). Fang et al. observed changes in the miRNA lineage in serum-derived EVs from depression-like rats that affect the function of key neurons in the brain, suggesting that they may be used as biomarkers to diagnose depression (Fang et al., 2020). Li et al. conducted miRNA sequencing on plasma-derived EVs from patients with depression and found that has-miR-335-5p was significantly upregulated, while has-miR-1292-3p was significantly downregulated (Li et al., 2021). These dysregulated miRNAs play an important role in axon formation and cell growth, providing a new possibility for the diagnosis of depression. Seki et al. employed miRNA chips to analyze miRNA expression levels in EVs from MDD patients, and the results indicated that the expressions of hsa-miR-6813-3p and hsa-miR-2277-3p were significantly downregulated as the severity of depression increased, demonstrating a strong correlation between the two (Seki et al., 2023). In addition, several researchers have identified proteins with potential diagnostic properties through proteomic analysis of EVs. For example, Jiang et al. showed that SERPINF1 can serve as a reliable biomarker for the development of MDD, and it is markedly reduced in depressed patients compared to healthy controls (Jiang et al., 2021). Zhang et al. discovered that the level of vitamin D-binding protein (VDBP) in plasma microglia-derived EVs from patients with depression was significantly reduced, a finding that was validated by the HAMD-24 score, suggesting that VDBP performed better as a diagnostic tool (Zhang G. et al., 2024).

Because of its stability and detectability, EVs provide new possibilities for diagnosing depression. However, although some related studies have been conducted in this field in recent years, there is still a lack of consensus on EVs as an available biomarker for depression, and it is necessary to expand the sample size and conduct systematic and comprehensive exploration.

### 3.3 Bipolar disorder (BD)

BD is a chronic and complex mental disorder characterized by intermittent or mixed episodes of depression and mania (Nierenberg et al., 2023). The pathogenesis of BD remains poorly understood, and definitive biomarkers are still lacking. Currently, BD diagnosis is primarily based on clinical observation and conversation, but its variable clinical manifestations often lead to misdiagnosis, highlighting the urgent need for biomarkers. A recent study found significant changes in expression levels of 33 miRNAs in EVs collected from patients with BD compared to healthy controls (Fries et al., 2019). Dysregulated miRNAs in BD are involved in the axon guidance of network proteins and the regulation of the serotonin receptor pathway, providing important preliminary evidence for the exploration of EVs as a biomarker of BD. Subsequently, Ceylan et al. found that compared to normal controls, miR-484, -652-3p, -142-3p in EVs of BD patients were significantly downregulated, while miR-185-5p was significantly upregulated (Ceylan et al., 2020). KEGG analysis revealed that dysregulated miRNAs participated in the pathological progression of BD through different target pathways such as PI3K/Akt signaling pathway, fatty acid biosynthesis/metabolism, extracellular matrix and adhesion pathway, which explained the importance of studying miRNAs from EVs as potential biomarkers in BD biomarker studies. Moreover, Du et al. analyzed metabolites in EVs from the serum of BD patients and identified 15 unbalanced metabolites. The ROC curve’s AUC value of 0.838 in the training set indicates that these metabolites may serve as diagnostic biomarkers for BD (Du et al., 2022). While the above studies provide evidence for the potential use of EVs in BD diagnosis, research in this area is still scarce. In the future, more researchers need to work together to analyze the complex causes of BD and identify more accurate biomarkers.

In addition, dysregulation of miRNA expression has also been observed in plasma-derived EVs from patients with autism, influencing the progression of neuropathology through various pathways (Chen et al., 2023; Qin et al., 2022). To sum up, the analysis of EVs collected from patients with mental disorders provides a wealth of information for the occurrence and development of diseases and the possibility for the diagnosis and process prediction of diseases (Table 1). Table 1 summarizes the potential biomarkers identified from EVs in various mental disorders, highlighting the valuable information they provide for disease progression and diagnosis. The transformation of EVs from laboratory research to clinical practice requires the joint efforts of researchers in the future.

**TABLE 1:** EVs as potential biomarkers for diagnosing mental disorders.

Disease	Source of EVs	Type of EVs	Biomarkers	References
Schizophrenia	Serum	Exosomes	hsa-miR-206	Du et al. (2019)
	Neuron	-	miRNA-1246 hsa-miR-451a	Barnett et al. (2023)
	Orbitofrontal cortex of brain	Exosomes	miR-223	Amoah et al. (2020)
	Blood	Exosomes	miR-137 cox - 6a2	Khadimallah et al. (2022)
	Plasma	Exosomes	miR-675-3p	Funahashi et al. (2023)
	Serum	Exosomes	25 metabolites	Du et al. (2021)
	Serum	Exosomes	lipids	Xu et al. (2024a)
	Plasma	Exosomes	GFAP α-II-Spectrin	Ranganathan et al. (2022)
	Plasma	Exosomes and microvesicles	NRGN, HPCA, KALRN, ADD2, ANK2	Tunset et al. (2020)
Major depression	Blood	Exosomes	hsa-miR-139-5p	Liang et al. (2020a), Wei et al. (2020)
	Serum	Exosomes	miRNA lineage	Fang et al. (2020)
	Plasma	Exosomes	has-miR-335-5p has-miR-1292-3p	Li et al. (2021)
	Serum	-	hsa-miR-6813-3p hsa-miR-2277-3p	Seki et al. (2023)
	Plasma	Exosomes	SERPINF1	Jiang et al. (2021)
	Microglia	Exosomes and microvesicles	VDBP	Zhang et al. (2024a)
Bipolar disorder	Plasma	-	miRNA lineage	Fries et al. (2019)
	Plasma	Exosomes	miR-484, miR-652-3p, miR-142-3p, miR-185-5p	Ceylan et al. (2020)
	Serum	Exosomes	15 metabolites	Du et al. (2022)
Autism	Plasma	-	miRNA lineage	Chen et al. (2023), Qin et al. (2022)

“-” represents EVs without detailed classification.

## 4 Application of EVs in the treatment of mental disorders

Due to their biocompatibility, stability, and low immunogenicity, EVs are becoming an attractive therapeutic strategy for CNS diseases (Li and Fang, 2023; Mattingly et al., 2021). The bimolecular lipid layer of EVs can protect their contents from protease attack, allowing them to remain stable in the extracellular environment and function over long distances. Additionally, EVs can penetrate various physiological barriers and target specific receptor cells through ligands on the membrane surface, thus achieving precise regulation. Recently, EVs-based treatments have begun to gain attention in addressing mental disorders.

### 4.1 EVs derived from mesenchymal stem cells

Previous studies have shown that the therapeutic effect of stem cells mainly depends on their paracrine function. Stem cell-derived EVs (MSC-EVs) can exert therapeutic effects in CNS diseases through various pathways such as neurogenesis, nerve regeneration, cognitive repair, and immune regulation (Harrell et al., 2021; Padinharayil et al., 2024). Li et al. established mouse models of depression and verified their therapeutic potential by intraperitoneal injection of EVs derived from human umbilical cord mesenchymal stem cells (hUC-MSCs). The results showed that EVs could inhibit the activation of M1 microglia and reduce neuroinflammation, thus exhibiting antidepressant effects (Li et al., 2024). Guo et al. constructed rat models of depression and treated them with EVs derived from bone marrow mesenchymal stem cells (Guo H. et al., 2020). These EVs upregulated the expression of miR-26a in the hippocampus of depressed rats, reduced the levels of malondialdehyde (MDA), lactate dehydrogenase (LDH), TNF-α and IL-1β, promoted the proliferation of hippocampal neurons, inhibited cell apoptosis, ultimately improving damage to hippocampal neurons in rats.

In recent years, intranasal administration, as a new non-invasive route of administration, has been gradually applied in the treatment of CNS diseases (Shen et al., 2023). Intranasal administration can bypass BBB and first-pass effects, enabling the drug to be rapidly enriched in the brain, with faster onset and higher bioavailability. Zhong et al. extracted EVs secreted from nasal olfactory mucosal mesenchymal stem cells (OM-MSCs-exos) (Zhong et al., 2024). After intranasal administration, OM-MSCs-exos inhibited the activation of microglia, alleviated neuroinflammation, and promoted nerve regeneration, thereby improving SCZ-like behavior. Tsivion-Visbord et al. found that intranasally administered MSC-EVs accumulated in the prefrontal cortex (PFC) of SCZ mice, alleviated the prepulse inhibition (PPI), enhanced neuronal viability, and had a positive effect on core SCZ-like behavior (Tsivion-Visbord et al., 2020).

Autism is a neurodevelopmental disorder characterized by social interaction defects, communication disorders, and stereotyped behavior. Studies have shown that MSC-EVs can positively influence autism-like behavior to a certain extent, increase social interaction and reduce repetitive behaviors (Liang Y. et al., 2020; Perets et al., 2020; Perets et al., 2018; Offen et al., 2019). After being administered intranasally, MSC-EVs can accumulate in the brain and continue to act for 72 h, effectively improving the behavioral phenotype of autism (Geffen et al., 2020b). Studies have found that MSC-EVs can affect the expression of immune regulation-related genes, alleviate neuroinflammation, and promote neurogenesis, thereby effectively improving autism-like behavior (Geffen et al., 2020a). In addition, MSC-EVs can significantly upregulate the expression of lncRNA Ifngas1 in the PFC of mice, activate the miR-21a-3p/PI3K/AKT signaling pathway, and promote neurogenesis, thereby exerting neuroprotective effects in autism mice (Fu et al., 2024).

Moreover, MSC-EVs have shown the ability to repair drug-induced nerve injury (Abdel-Gawad et al., 2024). Abdel-Gawad et al. demonstrated that the anti-tumor drug doxorubicin (DOX) can cause neuropathological changes in rats, leading to movement disorders and anxiety-like behaviors. However, after treatment with MSC-EVs, the pathological lesions and abnormal behavior in these animals caused by DOX can be significantly improved. To sum up, MSC-EVs can penetrate the brain, repair abnormal nerve damage, promote neurogenesis, improve the connection and communication between neurons, and show potential therapeutic effects in mental disorders.

### 4.2 EVs derived from microbial

Evidence suggests that microbes play a significant role in regulating host physiological functions, health, and disease development (Mitrea et al., 2022). Microbial-derived EVs can be taken up by host cells, and participate in signal transduction and regulation of biological processes, thereby affecting the physiological functions of the host. As a new therapeutic approach, microbial-derived EVs can transport neuroactive molecules produced by bacteria to the brain to function, which has attracted wide attention in the field of mental disorders (Guo et al., 2024). Choi et al. found that after treatment with Lactobacillus-derived EVs, the expressions of brain-derived neurotrophic factor (BDNF) and Nt4/5 in the hippocampus of depressed model mice were increased, and the depression-like behavior of mice was effectively improved (Choi et al., 2019). In addition, EVs secreted by Gram-positive probiotics, *Bacillus subtilis*, and Gram-negative probiotics, *Bacillus mucilaginous*, have also been validated to take antidepressant-like effects and play a positive role in counteracting changes in neurotrophic factors induced by chronic stress (Choi et al., 2022).

### 4.3 Engineering EVs

Due to their natural ability to cross various biological barriers, low immunogenicity, and modifiability, EVs have gained much attention in drug delivery. Functional EVs have been constructed by genetic engineering or chemical synthesis to improve the targeting and therapeutic efficacy of EVs (Schulze and Delalle, 2022). For instance, Lin et al. prepared EVs containing high levels of heat shock protein 70 (HSP70) by treating hUC-MSCs with heat shock (Lin et al., 2024). After treatment, they reduced the levels of TLR4 and p65 in the hippocampus of mice, regulated neuroinflammation and synaptic function, and significantly improved anxiety-like behavior and cognitive function in mice. Yu et al. designed and constructed an engineered EVs-drug delivery system, RVG-circDYM-ev, with enhanced brain targeting, which can effectively deliver circDYM to the brain. By binding with the transcription factor TATA-box binding protein-related factor 1 (TAF1), it reduces the expression of downstream target genes, inhibits neuroinflammation, and significantly improves depression-like behavior in mice (Yu et al., 2022).

In summary, EVs-based therapies have shown positive effects in the treatment of mental disorders (Table 2). However, the research of EVs in this field is still in the early stages, their specific mechanism of action is still unclear, and their actual efficacy remains to be further verified. In the future, more animal and clinical trials are necessary to explore the potential of EVs in mental disorders therapy.

**TABLE 2:** Application of EVs in the treatment of mental disorders.

Source	Types of EVs	Disease	Effects	References
Human umbilical cord mesenchymal stem cells (hUCMSCs)	Exosomes	Depression	Inhibit the activation of M1 microglia and reduce neuroinflammation	Li et al. (2024)
Bone marrow mesenchymal stem cells	Exosomes	Depression	Promote the proliferation of hippocampal neurons, inhibit apoptosis, and improve hippocampal neuron injury in rats	Guo et al. (2020a)
Nasal olfactory mucosal mesenchymal stem cells	Exosomes	Schizophrenia	Relieve neuroinflammation and promote nerve regeneration	Zhong et al. (2024)
Mesenchymal stem cells	-	Schizophrenia	Alleviate the prepulse inhibition of mice and enhance the neuronal activity	Tsivion-Visbord et al. (2020)
Mesenchymal stem cells	Exosomes	Autism	Improve ASD behavioral phenotype	Geffen et al. (2020b)
Mesenchymal stem cells	Exosomes	Autism	Aalleviate neuroinflammation	Geffen et al. (2020a)
Mesenchymal stem cells	Exosomes	Autism	Promote neurogenesis and protect nerves	Fu et al. (2024)
Mesenchymal stem cells	Exosomes	DOX-induced dyskinesia and anxiety	Repair abnormal nerve injury and promote neurogenesis	Abdel-Gawad et al. (2024)
*Lactobacillus*	-	Depression	Increase the expression of BNDF and Nt4/5 in the hippocampus of mice	Choi et al. (2019)
*Subtilis* and *mucilaginosus*	-	Depression	Antidepressant effect	Choi et al. (2022)
Heat shock treated hUCMSCs	-	Anxiety	Regulate neuroinflammation and synaptic function	Lin et al. (2024)
RVG-circDYM-ev	-	Depression	Inhibit neuroinflammation	Yu et al. (2022)

“-” represents EVs without detailed classification.

## 5 Conclusion

With the expansion and deepening of research, the potential roles of EVs in the occurrence, progression, diagnosis and treatment of mental disorders have been gradually revealed. In the normal physiological environment, EVs are the necessary medium for information exchange between cells in the brain, which is very important in maintaining CNS homeostasis. In contrast, in pathological conditions, EVs transmit pathological information between cells in the brain, induce neuroinflammation, and regulate disease progression. Pathological EVs can not only be found in the brain but can also cross the BBB and enter peripheral circulation. Researchers have extracted EVs from the peripheral blood of patients with schizophrenia, major depression, bipolar disorder and autism, as well as from healthy controls. The intrinsic components of EVs extracted from patients with mental disorders, such as nucleotides, proteins, lipids and metabolites, showed significant changes compared to those from healthy individuals. Based on these findings, EVs and their contents hold promise as biomarkers for diagnosing mental disorders. Moreover, due to their high stability, biocompatibility, low immunogenicity, and ability to cross the BBB, EVs are considered to be a promising and effective treatment strategy for mental disorders. In particular, mesenchymal stem cell-derived EVs have been found to effectively relieve neuroinflammation, restore neurogenesis, and promote neuroprotection, thus significantly improving the behavior and cognition of patients with mental disorders. In conclusion, EVs present broad application prospects for both the diagnosis and treatment of mental disorders.

However, the existing research on EVs is mainly in the laboratory stage, and it is still a great challenge to transform basic research into clinical applications. Firstly, the processes for extracting and detecting EVs are not standardized and can be easily affected by other substances in peripheral blood, resulting in insufficient yield and purity. Secondly, most existing studies focus on exosomes, while only a limited number of studies on complexes that have not been classified in detail, which lack effective comparison between different EVs types, and it remains unclear how different types and sizes of EVs may cause differences in effects. Futhermore, the origin of EVs is still uncertain, and the commonly used surface marker for brain-derived EVs, L1CAM, is now controversial, so more specific surface markers need to be found. Finally, the sample size of existing EVs-related experimental studies is small, and there may be differences between different studies. Therefore, whether EVs can become effective diagnostic markers or therapeutic agents for mental disorders requires more comprehensive research, including standardized isolation and characterization research, in-depth mechanism research, effective animal research, and large-scale clinical research.

## References

[B1] Abdel-Gawad D. R. I. Khalil F. Shehata O. Ibrahim M. A. El-Samannoudy S. Mahdi E. A. (2024). Role of bone marrow mesenchymal stem cell-derived exosomes in reducing neurotoxicity and depression-like behaviors induced by doxorubicin in rats. Toxicol. Res. (Camb) 13, tfae159. 10.1093/toxres/tfae159 39371677 PMC11447378

[B2] Ahmad S. Srivastava R. K. Singh P. Naik U. P. Srivastava A. K. (2022). Role of extracellular vesicles in glia-neuron intercellular communication. Front. Mol. Neurosci. 15, 844194. 10.3389/fnmol.2022.844194 35493327 PMC9043804

[B3] Amoah S. K. Rodriguez B. A. Logothetis C. N. Chander P. Sellgren C. M. Weick J. P. (2020). Exosomal secretion of a psychosis-altered miRNA that regulates glutamate receptor expression is affected by antipsychotics. Neuropsychopharmacology 45, 656–665. 10.1038/s41386-019-0579-1 31775160 PMC7021900

[B4] Arias D. Saxena S. Verguet S. (2022). Quantifying the global burden of mental disorders and their economic value. EClinicalMedicine 54, 101675. 10.1016/j.eclinm.2022.101675 36193171 PMC9526145

[B5] Arvanitaki E. S. Goulielmaki E. Gkirtzimanaki K. Niotis G. Tsakani E. Nenedaki E. (2024). Microglia-derived extracellular vesicles trigger age-related neurodegeneration upon DNA damage. Proc. Natl. Acad. Sci. U. S. A. 121, e2317402121. 10.1073/pnas.2317402121 38635632 PMC11047102

[B6] Bahram S. Gomes A. R. Curfs L. M. G. Reutelingsperger C. P. (2021). The role of Extracellular Vesicles during CNS development. Prog. Neurobiol. 205, 102124. 10.1016/j.pneurobio.2021.102124 34314775

[B7] Balu D. T. (2016). The NMDA receptor and schizophrenia: from pathophysiology to treatment. Adv. Pharmacol. 76, 351–382. 10.1016/bs.apha.2016.01.006 27288082 PMC5518924

[B8] Barnett M. M. Reay W. R. Geaghan M. P. Kiltschewskij D. J. Green M. J. Weidenhofer J. (2023). miRNA cargo in circulating vesicles from neurons is altered in individuals with schizophrenia and associated with severe disease. Sci. Adv. 9, eadi4386. 10.1126/sciadv.adi4386 38019909 PMC10686555

[B9] Bhom N. Somandi K. Ramburrun P. Choonara Y. E. (2025). Extracellular nanovesicles as neurotherapeutics for central nervous system disorders. Expert Opin. Drug Deliv. 22, 69–84. 10.1080/17425247.2024.2440099 39644485

[B10] Borroto-Escuela D. O. Pintsuk J. Schäfer T. Friedland K. Ferraro L. Tanganelli S. (2016). Multiple D2 heteroreceptor complexes: new targets for treatment of schizophrenia. Ther. Adv. Psychopharmacol. 6, 77–94. 10.1177/2045125316637570 27141290 PMC4837969

[B11] Borroto-Escuela D. O. Tarakanov A. O. Bechter K. Fuxe K. (2017). IL1R2, CCR2, and CXCR4 may form heteroreceptor complexes with NMDAR and D2R: relevance for schizophrenia. Front. Psychiatry 8, 24. 10.3389/fpsyt.2017.00024 28261115 PMC5309215

[B12] Ceylan D. Tufekci K. U. Keskinoglu P. Genc S. Özerdem A. (2020). Circulating exosomal microRNAs in bipolar disorder. J. Affect Disord. 262, 99–107. 10.1016/j.jad.2019.10.038 31726266

[B13] Chen R. Shi J. Yang H. Zhang M. Chen Q. He Q. (2023). Dysregulation of MicroRNAs derived from plasma extracellular vesicles in schizoaffective disorder. Mol. Neurobiol. 60, 6373–6382. 10.1007/s12035-023-03482-w 37452221

[B14] Chen Z. Li W. Meng B. Xu C. Huang Y. Li G. (2024). Neuronal-enriched small extracellular vesicles trigger a PD-L1-mediated broad suppression of T cells in Parkinson's disease. iScience 27, 110243. 10.1016/j.isci.2024.110243 39006478 PMC11246066

[B15] Choi J. Kim Y. K. Han P. L. (2019). Extracellular vesicles derived from Lactobacillus plantarum increase BDNF expression in cultured hippocampal neurons and produce antidepressant-like effects in mice. Exp. Neurobiol. 28, 158–171. 10.5607/en.2019.28.2.158 31138987 PMC6526105

[B16] Choi J. Kwon H. Kim Y. K. Han P. L. (2022). Extracellular vesicles from gram-positive and gram-negative probiotics remediate stress-induced depressive behavior in mice. Mol. Neurobiol. 59, 2715–2728. 10.1007/s12035-021-02655-9 35171438

[B17] Collaborators G. M. D. (2022). Global, regional, and national burden of 12 mental disorders in 204 countries and territories, 1990-2019: a systematic analysis for the Global Burden of Disease Study 2019. Lancet Psychiatry 9, 137–150. 10.1016/s2215-0366(21)00395-3 35026139 PMC8776563

[B18] Cornell J. Salinas S. Huang H. Y. Zhou M. (2022). Microglia regulation of synaptic plasticity and learning and memory. Neural Regen. Res. 17, 705–716. 10.4103/1673-5374.322423 34472455 PMC8530121

[B19] Crivelli S. M. Quadri Z. Vekaria H. J. Zhu Z. Tripathi P. Elsherbini A. (2023). Inhibition of acid sphingomyelinase reduces reactive astrocyte secretion of mitotoxic extracellular vesicles and improves Alzheimer's disease pathology in the 5xFAD mouse. Acta Neuropathol. Commun. 11, 135. 10.1186/s40478-023-01633-7 37605262 PMC10440899

[B20] Cserép C. Pósfai B. Dénes Á. (2021). Shaping neuronal fate: functional heterogeneity of direct microglia-neuron interactions. Neuron 109, 222–240. 10.1016/j.neuron.2020.11.007 33271068

[B21] Cui L. Li S. Wang S. Wu X. Liu Y. Yu W. (2024). Major depressive disorder: hypothesis, mechanism, prevention and treatment. Signal Transduct. Target Ther. 9, 30. 10.1038/s41392-024-01738-y 38331979 PMC10853571

[B22] Delaveris C. S. Wang C. L. Riley N. M. Li S. Kulkarni R. U. Bertozzi C. R. (2023). Microglia mediate contact-independent neuronal network remodeling via secreted neuraminidase-3 associated with extracellular vesicles. ACS Cent. Sci. 9, 2108–2114. 10.1021/acscentsci.3c01066 38033791 PMC10683476

[B23] Deng Y. Sun S. Wu S. Chen K. Liu Y. Wei W. (2024). Burden and trends of mental disorders in China from 1990 to 2019: findings from the global burden of disease study 2019. Soc. Psychiatry Psychiatr. Epidemiol. 59, 1563–1576. 10.1007/s00127-023-02594-x 38087123

[B24] Dong X. Jiang D. Wang L. Zhao J. Yu L. Huang Y. (2022). VPS28 regulates brain vasculature by controlling neuronal VEGF trafficking through extracellular vesicle secretion. iScience 25, 104042. 10.1016/j.isci.2022.104042 35330682 PMC8938284

[B25] Du Y. Chen L. Li X. S. Li X. L. Xu X. D. Tai S. B. (2021). Metabolomic identification of exosome-derived biomarkers for schizophrenia: a large multicenter study. Schizophr. Bull. 47, 615–623. 10.1093/schbul/sbaa166 33159208 PMC8084447

[B26] Du Y. Dong J. H. Chen L. Liu H. Zheng G. E. Chen G. Y. (2022). Metabolomic identification of serum exosome-derived biomarkers for bipolar disorder. Oxid. Med. Cell Longev. 2022, 5717445. 10.1155/2022/5717445 35047107 PMC8763519

[B27] Du Y. Yu Y. Hu Y. Li X. W. Wei Z. X. Pan R. Y. (2019). Genome-wide, integrative analysis implicates exosome-derived MicroRNA dysregulation in schizophrenia. Schizophr. Bull. 45, 1257–1266. 10.1093/schbul/sby191 30770930 PMC6811837

[B28] Durur D. Y. Tastan B. Ugur Tufekci K. Olcum M. Uzuner H. Karakülah G. (2022). Alteration of miRNAs in small neuron-derived extracellular vesicles of Alzheimer's disease patients and the effect of extracellular vesicles on microglial immune responses. J. Mol. Neurosci. 72, 1182–1194. 10.1007/s12031-022-02012-y 35488079

[B29] Faden J. Citrome L. (2023). Schizophrenia: one name, many different manifestations. Med. Clin. North Am. 107, 61–72. 10.1016/j.mcna.2022.05.005 36402500

[B30] Fan C. Li Y. Lan T. Wang W. Long Y. Yu S. Y. (2022). Microglia secrete miR-146a-5p-containing exosomes to regulate neurogenesis in depression. Mol. Ther. 30, 1300–1314. 10.1016/j.ymthe.2021.11.006 34768001 PMC8899528

[B31] Fang K. Xu J. X. Chen X. X. Gao X. R. Huang L. L. Du A. Q. (2020). Differential serum exosome microRNA profile in a stress-induced depression rat model. J. Affect Disord. 274, 144–158. 10.1016/j.jad.2020.05.017 32469797

[B32] Farizatto K. L. G. Baldwin K. T. (2023). Astrocyte-synapse interactions during brain development. Curr. Opin. Neurobiol. 80, 102704. 10.1016/j.conb.2023.102704 36913751

[B33] Filannino F. M. Panaro M. A. Benameur T. Pizzolorusso I. Porro C. (2024). Extracellular vesicles in the central nervous system: a novel mechanism of neuronal cell communication. Int. J. Mol. Sci. 25, 1629. 10.3390/ijms25031629 38338906 PMC10855168

[B34] Filippov V. Song M. A. Zhang K. Vinters H. V. Tung S. Kirsch W. M. (2012). Increased ceramide in brains with Alzheimer's and other neurodegenerative diseases. J. Alzheimers Dis. 29, 537–547. 10.3233/jad-2011-111202 22258513 PMC3643694

[B35] Fries G. R. Lima C. N. C. Valvassori S. S. Zunta-Soares G. Soares J. C. Quevedo J. (2019). Preliminary investigation of peripheral extracellular vesicles' microRNAs in bipolar disorder. J. Affect Disord. 255, 10–14. 10.1016/j.jad.2019.05.020 31125858

[B36] Fu Y. Zhang Y. L. Liu R. Q. Xu M. M. Xie J. L. Zhang X. L. (2024). Exosome lncRNA IFNG-AS1 derived from mesenchymal stem cells of human adipose ameliorates neurogenesis and ASD-like behavior in BTBR mice. J. Nanobiotechnology 22, 66. 10.1186/s12951-024-02338-2 38368393 PMC10874555

[B37] Funahashi Y. Yoshino Y. Iga J. I. Ueno S. I. (2023). Impact of clozapine on the expression of miR-675-3p in plasma exosomes derived from patients with schizophrenia. World J. Biol. Psychiatry 24, 303–313. 10.1080/15622975.2022.2104924 35904423

[B38] Gaebel W. Kerst A. Stricker J. (2020). Classification and diagnosis of schizophrenia or other primary psychotic disorders: changes from ICD-10 to ICD-11 and implementation in clinical practice. Psychiatr. Danub 32, 320–324. 10.24869/psyd.2020.320 33370728

[B39] Geffen Y. Horev R. Perets N. Marom E. Danon U. Offen D. (2020a). Immuno-Modulation and neuroprotection mediate the therapeutic effect of exosomes in mice model of autism. Cytotherapy 22, S49–S50. 10.1016/j.jcyt.2020.03.060

[B40] Geffen Y. Perets N. Horev R. Yudin D. Oron O. Elliott E. (2020b). Exosomes derived from adipose mesenchymal stem cells: a potential non-invasive intranasal treatment for autism. Cytotherapy 22, S49. 10.1016/j.jcyt.2020.03.059

[B41] González-Molina L. A. Villar-Vesga J. Henao-Restrepo J. Villegas A. Lopera F. Cardona-Gómez G. P. (2021). Extracellular vesicles from 3xTg-AD mouse and Alzheimer's disease patient astrocytes impair neuroglial and vascular components. Front. Aging Neurosci. 13, 593927. 10.3389/fnagi.2021.593927 33679370 PMC7933224

[B42] Guo C. Bai Y. Li P. He K. (2024). The emerging roles of microbiota-derived extracellular vesicles in psychiatric disorders. Front. Microbiol. 15, 1383199. 10.3389/fmicb.2024.1383199 38650872 PMC11033316

[B43] Guo H. Huang B. Wang Y. Zhang Y. Ma Q. Ren Y. (2020a). Bone marrow mesenchymal stem cells-derived exosomes improve injury of hippocampal neurons in rats with depression by upregulating microRNA-26a expression. Int. Immunopharmacol. 82, 106285. 10.1016/j.intimp.2020.106285 32088640

[B44] Guo M. Wang J. Zhao Y. Feng Y. Han S. Dong Q. (2020b). Microglial exosomes facilitate α-synuclein transmission in Parkinson's disease. Brain 143, 1476–1497. 10.1093/brain/awaa090 32355963 PMC7241957

[B45] Gutiérrez-Rojas L. Porras-Segovia A. Dunne H. Andrade-González N. Cervilla J. A. (2020). Prevalence and correlates of major depressive disorder: a systematic review. Braz J. Psychiatry 42, 657–672. 10.1590/1516-4446-2020-0650 32756809 PMC7678895

[B46] Harrell C. R. Volarevic A. Djonov V. Volarevic V. (2021). Mesenchymal stem cell-derived exosomes as new remedy for the treatment of neurocognitive disorders. Int. J. Mol. Sci. 22, 1433. 10.3390/ijms22031433 33535376 PMC7867043

[B47] Jauhar S. Johnstone M. Mckenna P. J. (2022). Schizophrenia. Lancet 399, 473–486. 10.1016/s0140-6736(21)01730-x 35093231

[B48] Jeon S. W. Kim Y. K. (2023). Neuron-microglia crosstalk in neuropsychiatric disorders. Adv. Exp. Med. Biol. 1411, 3–15. 10.1007/978-981-19-7376-5_1 36949303

[B49] Jiang M. Gu Y. F. Cai J. F. Wang A. He Y. Feng Y. L. (2021). MiR-186-5p dysregulation leads to depression-like behavior by de-repressing SERPINF1 in Hippocampus. Neuroscience 479, 48–59. 10.1016/j.neuroscience.2021.10.005 34648865

[B50] Jiao Z. He Z. Liu N. Lai Y. Zhong T. (2022). Multiple roles of neuronal extracellular vesicles in neurological disorders. Front. Cell Neurosci. 16, 979856. 10.3389/fncel.2022.979856 36204449 PMC9530318

[B51] Kaya Z. Belder N. Sever-Bahcekapili M. Donmez-Demir B. Erdener Ş E. Bozbeyoglu N. (2023). Vesicular HMGB1 release from neurons stressed with spreading depolarization enables confined inflammatory signaling to astrocytes. J. Neuroinflammation 20, 295. 10.1186/s12974-023-02977-6 38082296 PMC10712196

[B52] Khadimallah I. Jenni R. Cabungcal J. H. Cleusix M. Fournier M. Beard E. (2022). Mitochondrial, exosomal miR137-COX6A2 and gamma synchrony as biomarkers of parvalbumin interneurons, psychopathology, and neurocognition in schizophrenia. Mol. Psychiatry 27, 1192–1204. 10.1038/s41380-021-01313-9 34686767 PMC9054672

[B53] Kieling C. Buchweitz C. Caye A. Silvani J. Ameis S. H. Brunoni A. R. (2024). Worldwide prevalence and disability from mental disorders across childhood and adolescence: evidence from the global burden of disease study. JAMA Psychiatry 81, 347–356. 10.1001/jamapsychiatry.2023.5051 38294785 PMC10831630

[B54] Kong L. Zhang D. Huang S. Lai J. Lu L. Zhang J. (2023). Extracellular vesicles in mental disorders: a state-of-art review. Int. J. Biol. Sci. 19, 1094–1109. 10.7150/ijbs.79666 36923936 PMC10008693

[B55] Li B. Yang W. Ge T. Wang Y. Cui R. (2022). Stress induced microglial activation contributes to depression. Pharmacol. Res. 179, 106145. 10.1016/j.phrs.2022.106145 35219870

[B56] Li L. D. Naveed M. Du Z. W. Ding H. Gu K. Wei L. L. (2021). Abnormal expression profile of plasma-derived exosomal microRNAs in patients with treatment-resistant depression. Hum. Genomics 15, 55. 10.1186/s40246-021-00354-z 34419170 PMC8379796

[B57] Li P. Zhang F. Huang C. Zhang C. Yang Z. Zhang Y. (2024). Exosomes derived from DPA-treated UCMSCs attenuated depression-like behaviors and neuroinflammation in a model of depression induced by chronic stress. J. Neuroimmune Pharmacol. 19, 55. 10.1007/s11481-024-10154-6 39432176

[B58] Li Y. Fang B. (2023). Neural stem cell-derived extracellular vesicles: the light of central nervous system diseases. Biomed. Pharmacother. 165, 115092. 10.1016/j.biopha.2023.115092 37406512

[B59] Liang H. B. Chen X. Zhao R. Li S. J. Huang P. S. Tang Y. H. (2024). Simultaneous ischemic regions targeting and BBB crossing strategy to harness extracellular vesicles for therapeutic delivery in ischemic stroke. J. Control Release 365, 1037–1057. 10.1016/j.jconrel.2023.12.021 38109946

[B60] Liang J. Q. Liao H. R. Xu C. X. Li X. L. Wei Z. X. Xie G. J. (2020a). Serum exosome-derived miR-139-5p as a potential biomarker for major depressive disorder. Neuropsychiatr. Dis. Treat. 16, 2689–2693. 10.2147/ndt.S277392 33204094 PMC7667176

[B61] Liang Y. Duan L. Xu X. Li X. Liu M. Chen H. (2020b). Mesenchymal stem cell-derived exosomes for treatment of autism spectrum disorder. ACS Appl. Bio Mater 3, 6384–6393. 10.1021/acsabm.0c00831 35021769

[B62] Liang Y. Lehrich B. M. Zheng S. Lu M. (2021). Emerging methods in biomarker identification for extracellular vesicle-based liquid biopsy. J. Extracell. Vesicles 10, e12090. 10.1002/jev2.12090 34012517 PMC8114032

[B63] Lin Y. Kang Z. Su C. Li S. Xie W. (2024). Extracellular vesicles ameliorates sleep deprivation induced anxiety-like behavior and cognitive impairment in mice. Mol. Ther. Methods Clin. Dev. 32, 101207. 10.1016/j.omtm.2024.101207 38435131 PMC10907212

[B64] Liu X. Ying J. Wang X. Zheng Q. Zhao T. Yoon S. (2021). Astrocytes in neural circuits: key factors in synaptic regulation and potential targets for neurodevelopmental disorders. Front. Mol. Neurosci. 14, 729273. 10.3389/fnmol.2021.729273 34658786 PMC8515196

[B65] Liu Y. Shen X. Zhang Y. Zheng X. Cepeda C. Wang Y. (2023). Interactions of glial cells with neuronal synapses, from astrocytes to microglia and oligodendrocyte lineage cells. Glia 71, 1383–1401. 10.1002/glia.24343 36799296

[B66] Liu Y. J. Wang C. (2023). A review of the regulatory mechanisms of extracellular vesicles-mediated intercellular communication. Cell Commun. Signal 21, 77. 10.1186/s12964-023-01103-6 37055761 PMC10100201

[B67] Lizarraga-Valderrama L. R. Sheridan G. K. (2021). Extracellular vesicles and intercellular communication in the central nervous system. FEBS Lett. 595, 1391–1410. 10.1002/1873-3468.14074 33728650

[B68] Louka E. Koumandou V. L. (2024). The emerging role of human gut bacteria extracellular vesicles in mental disorders and developing new pharmaceuticals. Curr. Issues Mol. Biol. 46, 4751–4767. 10.3390/cimb46050286 38785554 PMC11120620

[B69] Mattingly J. Li Y. Bihl J. C. Wang J. (2021). The promise of exosome applications in treating central nervous system diseases. CNS Neurosci. Ther. 27, 1437–1445. 10.1111/cns.13743 34636491 PMC8611778

[B70] Mccutcheon R. A. Reis Marques T. Howes O. D. (2020). Schizophrenia-an overview. JAMA Psychiatry 77, 201–210. 10.1001/jamapsychiatry.2019.3360 31664453

[B71] Merrill R. M. Ashton M. K. (2024). How do mental disorders and combinations of disorders affect the odds of injuries and poisoning? J. Nerv. Ment. Dis. 212, 303–311. 10.1097/nmd.0000000000001771 38704650

[B72] Mitrea L. Nemeş S. A. Szabo K. Teleky B. E. Vodnar D. C. (2022). Guts imbalance imbalances the brain: a review of gut microbiota association with neurological and psychiatric disorders. Front. Med. (Lausanne) 9, 813204. 10.3389/fmed.2022.813204 35433746 PMC9009523

[B73] Nierenberg A. A. Agustini B. Köhler-Forsberg O. Cusin C. Katz D. Sylvia L. G. (2023). Diagnosis and treatment of bipolar disorder: a review. Jama 330, 1370–1380. 10.1001/jama.2023.18588 37815563

[B74] Offen D. Perets N. Oron O. Elliott E. Hertz S. London M. (2019). Treatment of mesenchymal stem cells derived exosomes leads to significant behavioral improvement of both genetic and idiopathic autismof mesenchymal stem cells derived exosomes leads to significant behavioral improvement of bothgenetic and idiopathic autism. Cytotherapy 21, E8. 10.1016/j.jcyt.2019.04.025

[B75] Padinharayil H. Varghese J. Wilson C. George A. (2024). Mesenchymal stem cell-derived exosomes: characteristics and applications in disease pathology and management. Life Sci. 342, 122542. 10.1016/j.lfs.2024.122542 38428567

[B76] Peng H. Harvey B. T. Richards C. I. Nixon K. (2021). Neuron-derived extracellular vesicles modulate microglia activation and function. Biol. (Basel) 10, 948. 10.3390/biology10100948 PMC8533634 34681047

[B77] Perets N. Hertz S. London M. Offen D. (2018). Intranasal administration of exosomes derived from mesenchymal stem cells ameliorates autistic-like behaviors of BTBR mice. Mol. Autism 9, 57. 10.1186/s13229-018-0240-6 30479733 PMC6249852

[B78] Perets N. Oron O. Herman S. Elliott E. Offen D. (2020). Exosomes derived from mesenchymal stem cells improved core symptoms of genetically modified mouse model of autism Shank3B. Mol. Autism 11, 65. 10.1186/s13229-020-00366-x 32807217 PMC7433169

[B79] Pullman L. E. Refaie N. Lalumière M. L. Krupp D. B. (2021). Is psychopathy a mental disorder or an adaptation? Evidence from a meta-analysis of the association between psychopathy and handedness. Evol. Psychol. 19, 14747049211040447. 10.1177/14747049211040447 34605282 PMC10358405

[B80] Qin Y. Cao L. Zhang J. Zhang H. Cai S. Guo B. (2022). Whole-transcriptome analysis of serum l1cam-captured extracellular vesicles reveals neural and glycosylation changes in autism spectrum disorder. J. Mol. Neurosci. 72, 1274–1292. 10.1007/s12031-022-01994-z 35412111

[B81] Raghavan V. Bhomia M. Torres I. Jain S. Wang K. K. (2017). Hypothesis: exosomal microRNAs as potential biomarkers for schizophrenia. Med. Hypotheses 103, 21–25. 10.1016/j.mehy.2017.04.003 28571801

[B82] Rahimian R. Wakid M. O'leary L. A. Mechawar N. (2021). The emerging tale of microglia in psychiatric disorders. Neurosci. Biobehav Rev. 131, 1–29. 10.1016/j.neubiorev.2021.09.023 34536460

[B83] Ramos-Zaldívar H. M. Polakovicova I. Salas-Huenuleo E. Corvalán A. H. Kogan M. J. Yefi C. P. (2022). Extracellular vesicles through the blood-brain barrier: a review. Fluids Barriers CNS 19, 60. 10.1186/s12987-022-00359-3 35879759 PMC9310691

[B84] Ranganathan M. Rahman M. Ganesh S. D'souza D. C. Skosnik P. D. Radhakrishnan R. (2022). Analysis of circulating exosomes reveals a peripheral signature of astrocytic pathology in schizophrenia. World J. Biol. Psychiatry 23, 33–45. 10.1080/15622975.2021.1907720 33821753

[B85] Richetin K. Steullet P. Pachoud M. Perbet R. Parietti E. Maheswaran M. (2020). Tau accumulation in astrocytes of the dentate gyrus induces neuronal dysfunction and memory deficits in Alzheimer's disease. Nat. Neurosci. 23, 1567–1579. 10.1038/s41593-020-00728-x 33169029

[B86] Schou A. S. Nielsen J. E. Askeland A. Jørgensen M. M. (2020). Extracellular vesicle-associated proteins as potential biomarkers. Adv. Clin. Chem. 99, 1–48. 10.1016/bs.acc.2020.02.011 32951635

[B87] Schulze T. G. Delalle I. (2022). Engineered extracellular vesicles and their promising therapeutic potential in neuropsychiatric disorders. Eur. Neuropsychopharmacol. 63, 14–16. 10.1016/j.euroneuro.2022.07.007 35914375

[B88] Seki I. Izumi H. Okamoto N. Ikenouchi A. Morimoto Y. Horie S. (2023). Serum extracellular vesicle-derived hsa-miR-2277-3p and hsa-miR-6813-3p are potential biomarkers for major depression: a preliminary study. Int. J. Mol. Sci. 24, 13902. 10.3390/ijms241813902 37762202 PMC10531403

[B89] Shen W. You T. Xu W. Xie Y. Wang Y. Cui M. (2023). Rapid and widespread distribution of intranasal small extracellular vesicles derived from mesenchymal stem cells throughout the brain potentially via the perivascular pathway. Pharmaceutics 15, 2578. 10.3390/pharmaceutics15112578 38004556 PMC10675165

[B90] Sung B. H. Parent C. A. Weaver A. M. (2021). Extracellular vesicles: critical players during cell migration. Dev. Cell 56, 1861–1874. 10.1016/j.devcel.2021.03.020 33811804 PMC8282723

[B91] Szepesi Z. Manouchehrian O. Bachiller S. Deierborg T. (2018). Bidirectional microglia-neuron communication in health and disease. Front. Cell Neurosci. 12, 323. 10.3389/fncel.2018.00323 30319362 PMC6170615

[B92] The Lancet P. (2024). Global Burden of Disease 2021: mental health messages. Lancet Psychiatry 11, 573. 10.1016/s2215-0366(24)00222-0 39025623

[B93] Théry C. Witwer K. W. Aikawa E. Alcaraz M. J. Anderson J. D. Andriantsitohaina R. (2018). Minimal information for studies of extracellular vesicles 2018 (MISEV2018): a position statement of the International Society for Extracellular Vesicles and update of the MISEV2014 guidelines. J. Extracell. Vesicles 7, 1535750. 10.1080/20013078.2018.1535750 30637094 PMC6322352

[B94] Tsivion-Visbord H. Perets N. Sofer T. Bikovski L. Goldshmit Y. Ruban A. (2020). Mesenchymal stem cells derived extracellular vesicles improve behavioral and biochemical deficits in a phencyclidine model of schizophrenia. Transl. Psychiatry 10, 305. 10.1038/s41398-020-00988-y 32873780 PMC7463024

[B95] Tunset M. E. Haslene-Hox H. Van Den Bossche T. Vaaler A. E. Sulheim E. Kondziella D. (2020). Extracellular vesicles in patients in the acute phase of psychosis and after clinical improvement: an explorative study. PeerJ 8, e9714. 10.7717/peerj.9714 32995075 PMC7501784

[B96] Van Niel G. Carter D. R. F. Clayton A. Lambert D. W. Raposo G. Vader P. (2022). Challenges and directions in studying cell-cell communication by extracellular vesicles. Nat. Rev. Mol. Cell Biol. 23, 369–382. 10.1038/s41580-022-00460-3 35260831

[B97] Wang H. He Y. Sun Z. Ren S. Liu M. Wang G. (2022a). Microglia in depression: an overview of microglia in the pathogenesis and treatment of depression. J. Neuroinflammation 19, 132. 10.1186/s12974-022-02492-0 35668399 PMC9168645

[B98] Wang J. Zhao Z. Yang J. Wang L. Zhang M. Zhou M. (2024). The association between depression and all-cause, cause-specific mortality in the Chinese population - China, 2010-2022. China CDC Wkly. 6, 1022–1027. 10.46234/ccdcw2024.212 39502121 PMC11532515

[B99] Wang Y. Amdanee N. Zhang X. (2022b). Exosomes in schizophrenia: pathophysiological mechanisms, biomarkers, and therapeutic targets. Eur. Psychiatry 65, e61. 10.1192/j.eurpsy.2022.2319 36082534 PMC9532215

[B100] Wei Z. X. Xie G. J. Mao X. Zou X. P. Liao Y. J. Liu Q. S. (2020). Exosomes from patients with major depression cause depressive-like behaviors in mice with involvement of miR-139-5p-regulated neurogenesis. Neuropsychopharmacology 45, 1050–1058. 10.1038/s41386-020-0622-2 31986519 PMC7162931

[B101] Welsh J. A. Goberdhan D. C. I. O'driscoll L. Buzas E. I. Blenkiron C. Bussolati B. (2024). Minimal information for studies of extracellular vesicles (MISEV2023): from basic to advanced approaches. J. Extracell. Vesicles 13, e12404. 10.1002/jev2.12404 38326288 PMC10850029

[B102] Willis C. M. Nicaise A. M. Bongarzone E. R. Givogri M. Reiter C. R. Heintz O. (2020). Astrocyte support for oligodendrocyte differentiation can be conveyed via extracellular vesicles but diminishes with age. Sci. Rep. 10, 828. 10.1038/s41598-020-57663-x 31964978 PMC6972737

[B103] Wilton D. K. Dissing-Olesen L. Stevens B. (2019). Neuron-glia signaling in synapse elimination. Annu. Rev. Neurosci. 42, 107–127. 10.1146/annurev-neuro-070918-050306 31283900

[B104] Woodburn S. C. Bollinger J. L. Wohleb E. S. (2021). The semantics of microglia activation: neuroinflammation, homeostasis, and stress. J. Neuroinflammation 18, 258. 10.1186/s12974-021-02309-6 34742308 PMC8571840

[B105] Wu Y. Wang L. Tao M. Cao H. Yuan H. Ye M. (2023). Changing trends in the global burden of mental disorders from 1990 to 2019 and predicted levels in 25 years. Epidemiol. Psychiatr. Sci. 32, e63. 10.1017/s2045796023000756 37933540 PMC10689059

[B106] Xian X. Cai L. L. Li Y. Wang R. C. Xu Y. H. Chen Y. J. (2022). Neuron secrete exosomes containing miR-9-5p to promote polarization of M1 microglia in depression. J. Nanobiotechnology 20, 122. 10.1186/s12951-022-01332-w 35264203 PMC8905830

[B107] Xiong Y. Mahmood A. Chopp M. (2024). Mesenchymal stem cell-derived extracellular vesicles as a cell-free therapy for traumatic brain injury via neuroprotection and neurorestoration. Neural Regen. Res. 19, 49–54. 10.4103/1673-5374.374143 37488843 PMC10479856

[B108] Xu C. X. Huang W. Shi X. J. Du Y. Liang J. Q. Fang X. (2024a). Dysregulation of serum exosomal lipid metabolism in schizophrenia: a biomarker perspective. Mol. Neurobiol. 62, 3556–3567. 10.1007/s12035-024-04477-x 39312067

[B109] Xu H. Li H. Zhang P. Gao Y. Ma H. Gao T. (2024b). The functions of exosomes targeting astrocytes and astrocyte-derived exosomes targeting other cell types. Neural Regen. Res. 19, 1947–1953. 10.4103/1673-5374.390961 38227520 PMC11040311

[B110] Xu X. Iqbal Z. Xu L. Wen C. Duan L. Xia J. (2024c). Brain-derived extracellular vesicles: potential diagnostic biomarkers for central nervous system diseases. Psychiatry Clin. Neurosci. 78, 83–96. 10.1111/pcn.13610 37877617

[B111] Yang C. Xue Y. Duan Y. Mao C. Wan M. (2024). Extracellular vesicles and their engineering strategies, delivery systems, and biomedical applications. J. Control Release 365, 1089–1123. 10.1016/j.jconrel.2023.11.057 38065416

[B112] Yin T. Liu Y. Ji W. Zhuang J. Chen X. Gong B. (2023). Engineered mesenchymal stem cell-derived extracellular vesicles: a state-of-the-art multifunctional weapon against Alzheimer's disease. Theranostics 13, 1264–1285. 10.7150/thno.81860 36923533 PMC10008732

[B113] You Y. Borgmann K. Edara V. V. Stacy S. Ghorpade A. Ikezu T. (2020). Activated human astrocyte-derived extracellular vesicles modulate neuronal uptake, differentiation and firing. J. Extracell. Vesicles 9, 1706801. 10.1080/20013078.2019.1706801 32002171 PMC6968484

[B114] Yu X. Bai Y. Han B. Ju M. Tang T. Shen L. (2022). Extracellular vesicle-mediated delivery of circDYM alleviates CUS-induced depressive-like behaviours. J. Extracell. Vesicles 11, e12185. 10.1002/jev2.12185 35029057 PMC8758833

[B115] Zhang G. Li L. Kong Y. Xu D. Bao Y. Zhang Z. (2024a). Vitamin D-binding protein in plasma microglia-derived extracellular vesicles as a potential biomarker for major depressive disorder. Genes Dis. 11, 1009–1021. 10.1016/j.gendis.2023.02.049 37692510 PMC10491883

[B116] Zhang J. Shi W. Qu D. Yu T. Qi C. Fu H. (2022). Extracellular vesicle therapy for traumatic central nervous system disorders. Stem Cell Res. Ther. 13, 442. 10.1186/s13287-022-03106-5 36056445 PMC9438220

[B117] Zhang X. Alnafisah R. S. Hamoud A. A. Shukla R. Wen Z. Mccullumsmith R. E. (2021). Role of astrocytes in major neuropsychiatric disorders. Neurochem. Res. 46, 2715–2730. 10.1007/s11064-020-03212-x 33411227

[B118] Zhang X. M. Huang J. Ni X. Y. Zhu H. R. Huang Z. X. Ding S. (2024b). Current progression in application of extracellular vesicles in central nervous system diseases. Eur. J. Med. Res. 29, 15. 10.1186/s40001-023-01606-5 38173021 PMC10763486

[B119] Zhong X. L. Huang Y. Du Y. He L. Z. Chen Y. W. Cheng Y. (2024). Unlocking the therapeutic potential of exosomes derived from nasal olfactory mucosal mesenchymal stem cells: restoring synaptic plasticity, neurogenesis, and neuroinflammation in schizophrenia. Schizophr. Bull. 50, 600–614. 10.1093/schbul/sbad172 38086528 PMC11059802

